# Comorbid Depression and Heart Failure: A Community Cohort Study

**DOI:** 10.1371/journal.pone.0158570

**Published:** 2016-06-30

**Authors:** Bhautesh Dinesh Jani, Frances S. Mair, Véronique L. Roger, Susan A. Weston, Ruoxiang Jiang, Alanna M. Chamberlain

**Affiliations:** 1 General Practice and Primary Care, Institute of Health and Wellbeing, University of Glasgow, Glasgow, United Kingdom; 2 Department of Health Sciences Research, Mayo Clinic, Rochester, Minnesota, United States of America; 3 Division of Cardiovascular Diseases, Mayo Clinic, Rochester, Minnesota, United States of America; Tokai University, JAPAN

## Abstract

**Objective:**

To examine the association between depression and clinical outcomes in heart failure (HF) in a community cohort.

**Patients and Methods:**

HF patients in Minnesota, United States completed depression screening using the 9-item Patient Health Questionnaire (PHQ-9) between 1^st^ Oct 2007 and 1^st^ Dec 2011; patients with PHQ-9≥5 were labelled “depressed”. We calculated the risk of death and first hospitalization within 2 years using Cox regression. Results were adjusted for 10 commonly used prognostic factors (age, sex, systolic blood pressure, estimated glomerular filtration rate, serum sodium, ejection fraction, blood urea nitrogen, brain natriuretic peptide, presence of diabetes and ischaemic aetiology). Area under the curve (AUC), integrated discrimination improvement (IDI) and net reclassification improvement (NRI) compared depression as a predictor against the aforementioned factors.

**Results:**

425 patients (mean age 74, 57.6% males) were included in the study; 179 (42.1%) had PHQ-9≥5. The adjusted hazard ratio of death was 2.02 (95% CI 1.34–3.04) and of hospitalization was 1.42 (95% CI 1.13–1.80) for those with compared to those without depression. Adding depression to the models did not appreciably change the AUC but led to statistically significant improvements in both the IDI (p = 0.001 and p = 0.005 for death and hospitalization, respectively) and NRI (for death and hospitalization, 35% (p = 0.002) and 27% (p = 0.007) were reclassified correctly, respectively).

**Conclusion:**

Depression is frequent among community patients with HF and associated with increased risk of hospitalizations and death. Risk prediction for death and hospitalizations in HF patients can be improved by considering depression.

## Introduction

Heart failure (HF) is a major health problem with high rates of mortality and hospitalization reported across Europe and North America [1–3]. Accurate prediction of prognosis in chronic HF patients is important for decision making and helps identify patients at risk who may benefit from closer monitoring [4,5]. Various risk prediction models have been proposed to predict mortality and hospitalization in HF [6–11]. A recently published systematic review by Ouwerkerk and colleagues has identified 11 of the most commonly used prognostic markers in the literature for risk prediction of chronic HF outcomes [6]. However, there are a number of drawbacks with currently available prognostic models, such as limited accuracy and scarcity of data available on predicting hospitalisation; hence, better prognostic markers are required for HF patients [7–10].

Depression has been found to be an independent predictor of mortality and hospitalization in HF [12–16]. However, the clinical utility of depression as a prognostic marker for HF outcomes has not been examined in comparison with some of the commonly used HF prognostic markers. Thus, the objective of this study was to examine if the presence of co-morbid depression provided incremental prognostic information for 2-year mortality and hospitalization risk prediction over the most commonly used prognostic markers in HF[6].

## Methods

### Study Setting

This was a prospective cohort study conducted in southeast Minnesota (with an approximate population of 185,000) [17] in the United States using the Rochester Epidemiology Project [18–20], a record linkage system which allows near complete capture of health care utilization for area residents. This is possible because the area is relatively isolated from other urban centers and has a small number of medical providers, including Mayo Clinic and Olmsted Medical Center, which deliver nearly all health care to local residents. This study was approved by the Mayo Clinic and Olmsted Medical Center Institutional Review Boards.

### Study Sample

Patients with either incident or prevalent HF were identified during an inpatient or outpatient visit using natural language processing of the electronic medical record. The diagnoses were manually validated by trained abstractors using the Framingham criteria, which has been described previously [14]. HF patients aged ≥18 years who resided in Olmsted, Dodge or Fillmore Counties in Minnesota were prospectively recruited between October 2007 and December 2011 and asked to complete a 9-item Patient Health Questionnaire (PHQ-9) [21] for depression. Written informed consent was obtained from all participants.

### Depression Assessment

Depression symptoms were assessed using the PHQ-9 administered by a registered nurse in person, within 6 weeks of enrolment. A PHQ-9 score of 5 or more denotes mild depression, while a score of 10 or more is indicative of major depressive disorder [21]. Hence, a score of PHQ-9 ≥5 was used to define “presence of depressive symptoms”, while a score of PHQ-9≥10 was used to define “presence of moderate to severe depression”. All clinical variables were either obtained electronically or from patient records.

### Measurement of Clinical Variables

Systolic blood pressure in mm Hg was obtained within 30 days of recording PHQ-9. Estimated glomerular filtration rate (eGFR) was estimated using the closest serum creatinine value within 1 year of administering PHQ-9 using the Modification of Diet in Renal Disease Study equation [22]. Left ventricular ejection fraction (EF) measured in % was obtained using the closest value from an echocardiogram within 6 months prior to 2 months after the patient’s diagnosis of HF (inpatient or outpatient) that identified them for recruitment into our study. Serum sodium (measured in mmol/l), blood urea nitrogen (measured in mg/dl), B-Type natriuretic peptide (BNP) (measured in pg/ml) and N-Terminal pro-BNP (NT-BNP) (measured in pg/ml) values were obtained within 1 year of administering PHQ-9. BNP values were used only when NT-BNP values were not available. Because of the need to use both BNP and NT-BNP data, we dichotomized them into raised vs. not raised. Raised BNP was defined as values more than 400 pg/ml. Raised NT-BNP was defined as values more than 450 pg/ml for age<50, more than 900 pg/ml for age 50–75, and more than 1800 pg/ml for age >75 [23].

### Measurement of Clinical Outcomes

Participants were followed for 2 years after administering PHQ-9 for all-cause death and all-cause hospitalization. Deaths were obtained from inpatient and outpatient medical records and death certificates received from the state of Minnesota. Hospitalizations were ascertained using data from the Rochester Epidemiology Project. For patients hospitalized at the time of their HF, only subsequent hospitalizations were included in the analysis. In-hospital transfers or transfers between Olmsted Medical Center and Mayo Clinic were analysed as a single hospitalization event.

### Statistical Analysis

Baseline patient characteristics were reported as a frequency (%) for categorical variables and mean (standard deviation [SD]) or median (25^th^ percentile, 75^th^ percentile) for continuous variables. Two-sample t-tests and χ^2^ tests were used to test differences in baseline characteristics between patients with and without depressive symptoms for continuous and categorical variables, respectively. A Kaplan-Meier survival plot was constructed to illustrate the association between depression and all-cause mortality. A cumulative incidence plot was constructed for first hospitalization treating death as a competing risk. Cox proportional hazards regression models were used to examine the associations between presence of depressive symptoms with all-cause mortality and first hospitalization. The proportional hazards assumption was tested for both outcomes and found to be valid. Results were reported as hazard ratios (HR) with 95% confidence intervals (CI).

Ten of the 11 most commonly used prognostic markers for chronic HF outcomes identified from the published literature by Ouwerkerk and his colleagues were included in the model as confounding factors [6]; we chose this model as it distinguishes between prognostic markers for acute and chronic HF patients. Information on the New York Heart Association (NYHA) functional classification [24] was not consistently available and thus was not included in the model. Age, systolic blood pressure, estimated glomerular filtration rate, serum sodium and blood urea nitrogen were included in multivariable models as confounders and modelled as continuous variables. Ejection fraction was log transformed and included as continuous variables in the multivariable models. Gender, presence of diabetes, ischaemic aetiology and elevated B-Type natriuretic peptide (BNP) or N-Terminal pro-BNP (NT-BNP) were included as categorical variables. The 10 confounding factors identified by Ouwerkerk and colleagues were included in all multivariable models.

The prognostic utility of presence of depressive symptoms for 2-year mortality and hospitalization risk prediction was compared against a base model consisting of the 10 prognostic markers described above using three different statistical methods: area under the receiver operating characteristic curve (AUC), integrated discrimination improvement (IDI) and a continuous version of the net reclassification improvement (NRI) [25,26]. The IDI indicates if adding presence of depressive symptoms to the prediction model improves the discrimination slope, defined as the average predicted probability of outcome for those who experienced the outcome versus those who did not. The IDI is the difference in the discrimination slopes for the models with and without presence of depressive symptoms. The NRI assesses net improvement in risk classification. Individuals are divided into those who experienced the outcome and those who did not. The predicted probability of the outcome is calculated for each individual, first using the base prediction model and then after adding presence of depressive symptoms to the model. The NRI is a measure of the number of individuals who experienced the outcome who were reclassified upward and the number of individuals who did not experience the outcome who were reclassified downward after adding presence of depressive symptoms to the model. Outcomes within the first 2 years after HF were included in the analyses. Because the NRI and IDI analyses require that the outcome be known, patients who were lost to follow-up before 2 years and who were known to be alive at the last follow-up were excluded from the analyses. In predicting all-cause mortality and hospitalization, values of AUC were reported for the base model and after adding presence of depressive symptoms. A p-value of less than 0.05 was used to assess statistical significance. Sensitivity analyses included repeating the analyses for the presence of moderate to severe depression (PHQ-9≥10), and also repeating the analysis using PHQ-9 as a continuous variable. All analyses were performed using R 3.0.2 (The R Foundation for Statistical Computing) [27] and SAS version 9.3 (SAS Institute Inc., Cary, NC).

## Results

### Patient Population and Characteristics

**Fig 1:**
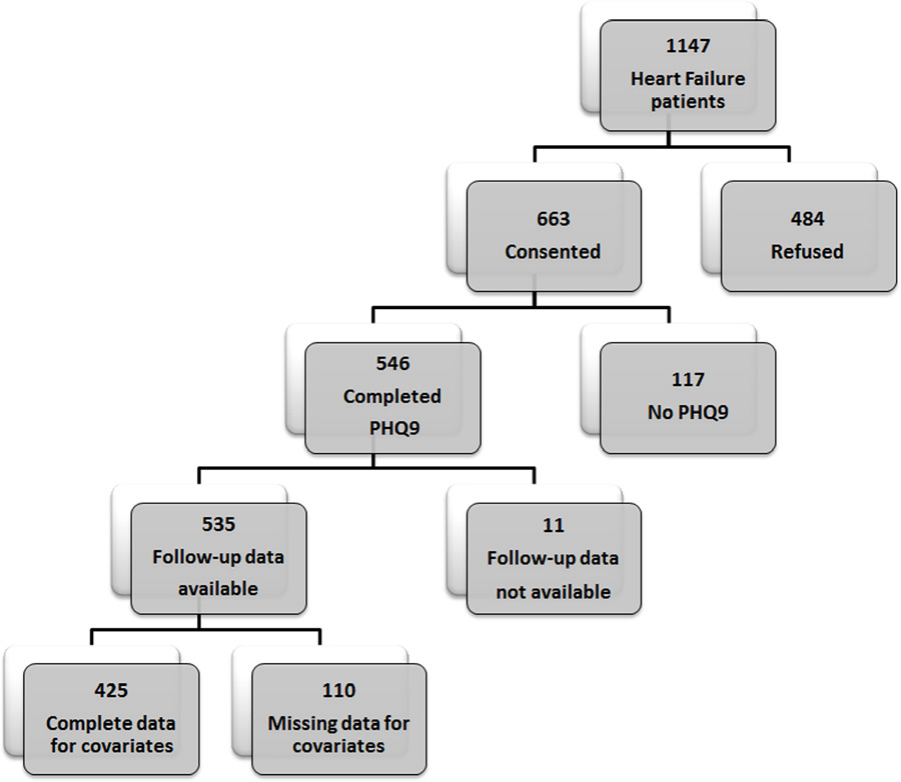
Patient Recruitment. PHQ-9 = 9-item Patient Health Questionnaire.

A total of 1147 patients with chronic HF diagnosed between October 2007 and December 2011 were approached to participate in the study and 663 (58.0%) patients consented. The patients who were approached but refused to participate were significantly older (mean age 78.6 ± 12.2 vs. 74.1 ± 13.3, respectively, p<0.001) and significantly more likely to be female (54.3% vs 45.4% respectively, p = 0.003) than study participants. Of those, 546 (82.0%) completed the PHQ-9 at a median time of 39 days (25^th^, 75^th^ percentile: 27, 58) after enrolment. Eleven patients were excluded because they were lost to follow-up before the end of 2 years and 110 were excluded due to missing covariate values, resulting in 425 patients (mean age (SD) 73.5 (13.2); 57.6% men) included in the analyses (Fig 1). Among the study participants, 179 (42.1%) patients had depressive symptoms based on a PHQ-9 score ≥5, while 61 (14.4%) patients were classified as having moderate to severe depression based on a PHQ-9 score ≥10. The 10 clinical measures included in the base prognostic model are presented for patients with and without depressive symptoms in Table 1. No statistically significant differences were observed between the two patient groups, with the exception of age as patients with depressive symptoms were younger.

**Table 1:** Established prognostic factors at baseline in chronic heart failure patients with and without depressive symptoms.

	Patients with depressive symptoms (N = 179)	Patients without depressive symptoms (N = 246)	p-value
Age (years), mean (SD)	71.77 (13.50)	74.79 (12.77)	**0.02**
Male	108 (60.34%)	137 (55.69%)	0.34
Systolic BP (mm Hg), mean(SD)	123.00 (23.12)	124.57 (23.04)	0.49
Estimated glomerular filtration rate, mean (SD)	56.32 (25.29)	57.70 (21.16)	0.54
Ejection fraction (%), median (25th, 75th percentile)	45.33 (31.00, 60.00)	50.00 (33.20, 60.00)	0.26
Serum sodium (mmol/l), median (25^th^, 75^th^ percentile)	140.00 (137.00,141.00)	139.00 (137.00,141.00)	0.88
Elevated level of BNP/NT-BNP	126 (70.39%)	177 (71.95%)	0.73
Ischemic etiology	76 (42.46%)	105 (42.68%)	0.96
Prior diabetes mellitus	76 (42.46%)	87 (35.37%)	0.14

Legend: Results are reported as n (%) unless otherwise noted. Presence of depressive symptoms defined as 9-item Patient Health Questionnaire (PHQ-9) ≥5. BNP = B-Type natriuretic peptide; BP = blood pressure; NT-BNP = N-Terminal pro-BNP; PHQ-9 = 9-item Patient Health Questionnaire; SD = standard deviation.

### Presence of Depressive Symptoms, All-cause Mortality and Hospitalization

**Fig 2:**
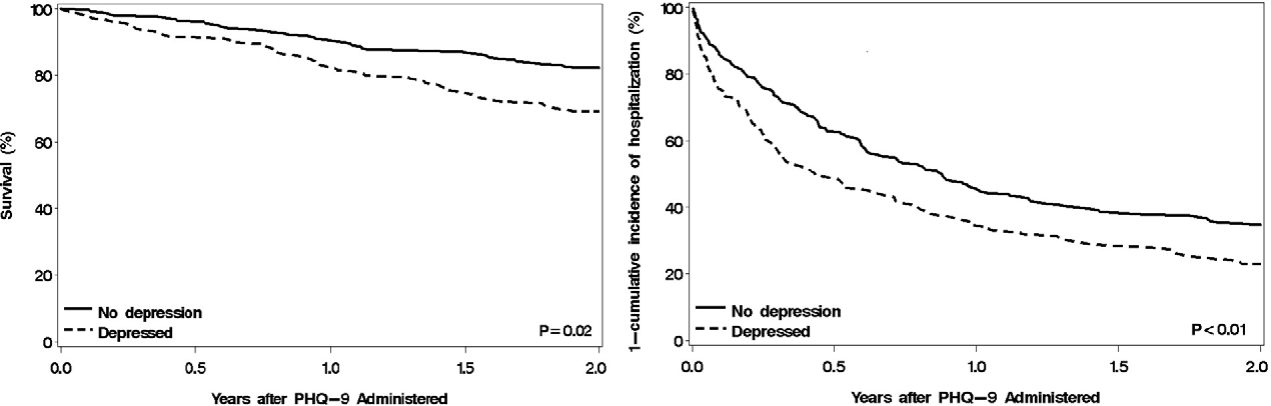
**(A) Survival Plot for All-cause Mortality for Chronic Heart Failure Patients. (B) 1-Cumulative Incidence Plot for First Hospitalization Treating Death as a Competing Risk for Chronic Heart Failure Patients.** Depressed Defined as 9-item Patient Health Questionnaire (PHQ-9) ≥5.

At the end of 2 years, 99 (23.3%) patients had died and 299 (70.4%) patients had at least 1 hospitalization. Patients with depressive symptoms had worse survival and hospitalization-free survival over 2 years of follow-up (Fig 2). Presence of depressive symptoms was associated with more than a 2-fold increased risk of all-cause mortality within 2 years, unadjusted and after adjusting for the 10 most commonly used prognostic factors (Table 2). The unadjusted and adjusted risk of hospitalization was almost 50% higher among HF patients with depression compared to those without it.

**Table 2:** Hazard ratios for all-cause death and first hospitalization within 2 years after HF for chronic heart failure patients with vs without depressive symptoms.

	All-Cause Death	Hospitalization
Number of patients	425	425
Number of events	99	299
Unadjusted HR (95% CI)	1.87 (1.26, 2.78)	1.48 (1.18, 1.86)
Adjusted* HR (95% CI)	2.02 (1.34–3.04)	1.42 (1.13–1.80)

Legend: Presence of depressive symptoms defined as 9-item Patient Health Questionnaire (PHQ-9) ≥5. CI = Confidence interval; HR = Hazard Ratio.

*Adjusted for age, sex, systolic blood pressure, estimated glomerular filtration rate, blood urea nitrogen, serum sodium, elevated B-Type natriuretic peptide (BNP) or N-Terminal pro-BNP, ejection fraction, ischaemic aetiology and prior diabetes.

### Prognostic Utility of Depression in Prediction of All-cause Mortality and First Hospitalization

Table 3 compares the prognostic utility of adding depressive symptoms to the base model in predicting all-cause mortality and hospitalization within 2 years. The difference between the two models was not statistically significant for AUC values for either of the two outcomes. However, the IDI and NRI values showed statistically significant improvement for predicting all-cause mortality after adding depressive symptoms. Regarding hospitalizations, the IDI and NRI-continuous showed statistically significant improvement after adding depressive symptoms to the model.

**Table 3:** Comparison of the prognostic utility of adding depressive symptoms to the base model in predicting all-cause mortality and hospitalization within 2 years after heart failure in chronic heart failure patients.

Outcome	Model	AUC (95% CI)	IDI, % (95% CI)	NRI-continuous, % (95% CI)
All-cause Death	Base model*	0.781(0.729–0.834)		
	Base model + depressive symptoms	0.800(0.748–0.852)	3.10(1.28–4.92)	35.04(12.80–57.27)
	p-value	0.06	0.001	0.002
Hospitalization	Base model*	0.667(0.609–0.724)		
	Base model + depressive symptoms	0.679(0.621–0.736)	1.73(0.53–2.93)	27.23(7.34–47.11)
	p-value	0.36	0.005	0.007

Legend: Presence of depressive symptoms defined as 9-item Patient Health Questionnaire (PHQ-9) ≥5. AUC = area under curve; CI = confidence interval; IDI = integrated discrimination improvement; NRI = net re-classification improvement.

*Base model includes age, sex, systolic blood pressure, estimated glomerular filtration rate, blood urea nitrogen, serum sodium, elevated B-Type natriuretic peptide (BNP) or N-Terminal pro-BNP, ejection fraction, ischaemic aetiology and prior diabetes.

### Sensitivity Analysis

Results of sensitivity analyses are presented in S1 File. A stronger association was observed between presence of moderate to severe depression (PHQ-9≥10) and all-cause mortality and hospitalization, when compared to mild or no depressive symptoms. Additionally, the NRI and IDI values improved significantly in predicting death and hospitalization, while there was no significant change in the AUC values Furthermore, when analysing the PHQ-9 score as a continuous variable, a 10% increase in all-cause death and a 5% increase in hospitalization were observed per point increase in PHQ-9 score after adjustment for the 10 most commonly used prognostic factors.

## Discussion

In a community cohort in the US, patients with chronic HF were found to have a high prevalence of depressive symptoms. Depression was associated with a higher risk of death and hospitalization compared to those without depression. These findings remain unchanged after adjusting for the 10 most commonly used prognostic factors in risk prediction for HF outcomes. Finally, adding depression to an existing prognostic model improved the prognostic utility in predicting death and hospitalization.

Published results on the prevalence of depression in HF are varied. In our cohort, the prevalence of depression was 40.7% based on a symptom questionnaire, which is congruent with the reported prevalence of 33.6% in a meta-analysis [15]. HF patients with co-existing depression were approximately twice as likely to die in our study, which is consistent with previous findings [15]. Depression was also associated with an increase in the risk of hospitalization, which is again consistent with previous findings [14,15,28–31].

Two previous studies have assessed the prognostic utility of depression; however they have used “history of previous depression” as opposed to “current depression” as was used in our study [32,33]. Herein, addition of depression did not improve AUC values from the base model for predicting death and hospitalization. The lack of sensitivity of AUC in judging prognostic utility of a new marker has been discussed previously and the present study underscores the importance of incorporating methods such as IDI and NRI in risk prediction [26,34].

### Limitations, Strengths and Implications

Depressive symptoms were measured only at enrolment and we cannot account for changes during follow-up. Some of the symptoms of depression overlap with common symptoms of HF, such as fatigue, low energy, psychomotor retardation, and sleep disturbances [13]. While the NYHA functional status was not consistently available in our cohort, evidence suggests inconsistency and high inter-operator variability in clinical recordings of NYHA in practice, which illustrates the practical problems in using it as a prognostic marker [35]. Further, depression has been shown to predict death and hospitalization in HF independent of NYHA functional status [28]. Finally, the population of southeast Minnesota is chiefly white and thus, our results should be examined in other racial groups.

Our study has a number of notable strengths. The participants were recruited from a community cohort, including both inpatients and outpatients, which is of optimal clinical relevance. Depression was prospectively ascertained using a validated instrument and follow-up was complete for critical outcomes in HF. Analytically, we used robust and complementary risk prediction methods which optimize our ability to assess the prognostic value of depression.

Despite the high prevalence of depressive symptoms in HF [15], it remains under recognized [36], and no study to date, to the best of our knowledge, has demonstrated the benefits of routine depression screening [37]. Indeed, treatment with anti-depressants has not shown any clear benefit in reducing depressive symptoms, deaths and hospitalization in HF [38–40]. There is some conflicting evidence about the use of cognitive behavioural therapy (CBT) in reducing depressive symptoms in HF patients, with a review suggesting no benefit [41], while some of the recent studies showing improvement in depressive symptoms with CBT [42,43]. On the other hand, other psychological interventions such as mindfulness-based stress reduction [44] have been shown to have the potential to improve depressive symptoms in HF patients. The recent guidelines published by the American Heart Association and the American College of Cardiology guidelines discuss the importance of depression as an important co-morbidity in heart failure patients and its association with reduced poor quality of life and poor health outcomes [4]. Some research suggests that lack of perceived social support may be an important mediator of poor prognosis associated with depression in HF patients [45,46],which in turn is potentially modifiable [47]. Further research should address these knowledge gaps.

It is important to underscore that ascertaining depression relies on a clinical assessment, which is efficient and not costly. We demonstrated the incremental information conferred by depression over well-established clinical factors, thereby indicating that assessing mental health and depression should be part of the holistic clinical evaluation of patients living with HF.

## Conclusion

In HF, depression is frequent and is associated with an increase in deaths and hospitalizations. Depression increases the prognostic value of established and commonly used factors in HF patients. Further research is needed to determine the role of depression screening and ascertain the best strategies for managing depressive symptoms in HF patients.

## Supporting Information

S1 File Sensitivity Analysis. (DOCX)

S2 File Dataset. (XLSX)

## Abbreviations

HF: Heart Failure
PHQ-9: Patient Health Questionnaire
AUC: Area Under Curve
NRI: Net Reclassification Improvement
IDI: Integrated Discrimination Improvement
HR: Hazard Ratio
CI: Confidence Intervals
