# Supplementary material for: Comorbid Depression and Heart Failure: A Community Cohort Study
Source: PLoS One. 2016 Jun 30;11(6):e0158570. doi: 10.1371/journal.pone.0158570 (PMC4928788; doi:10.1371/journal.pone.0158570)
Supplement: S1 File — (DOCX) [file pone.0158570.s001.docx]

**Supplement- Sensitivity Analysis**

**Part 1: Presence of moderate to severe depressive symptoms (PHQ-9≥10) as a prognostic marker in HF patients**

**Table S1**

Established prognostic factors at baseline in chronic heart failure patients with and without moderate to severe depressive symptoms.

|  | Patients with moderate to severe depression  (N=61) | Patients without moderate to severe depression  (N=364) | p-value |
| --- | --- | --- | --- |
| Age (years), mean (SD) | 70.49 (13.08) | 74.02 (13.12) | 0.05 |
| Male | 32 (52.46%) | 213 (58.52%) | 0.38 |
| Systolic BP (mm Hg), mean (SD) | 123.44 (21.09) | 123.99 (23.40) | 0.86 |
| Estimated Glomerular Filtration Rate, mean (SD) | 59.78 (29.58) | 56.67 (21.69) | 0.33 |
| Ejection fraction (%), median (25^th^, 75^th^ percentile) | 50.67  (31.00, 62.00) | 48.75  (32.75, 60.00) | 0.94 |
| Serum sodium (mmol/l), median (25^th^, 75^th^ percentile) | 140.00 (137.00,141.00) | 139.00 (137.00,141.00) | 0.71 |
| Blood urea nitrogen (mg/dl), median (25^th^, 75^th^ percentile) | 29.00  (19.00,42.00) | 26.00  (19.00,36.00) | 0.61 |
| Elevated level of BNP/NT-BNP | 44 (72.13%) | 259 (71.15%) | 0.88 |
| Ischemic etiology | 28 (45.90%) | 153 (42.03%) | 0.57 |
| Prior diabetes mellitus | 29 (47.54%) | 134 (36.81%) | 0.11 |

Results are reported as n (%) unless otherwise noted.

Moderate to severe depression defined as 9-item Patient Health Questionnaire (PHQ-9) ≥10.

BNP=B-Type natriuretic peptide; BP=blood pressure; NT-BNP=N-Terminal pro-BNP; PHQ-9= 9-item Patient Health Questionnaire; SD=standard deviation

**Table S2**

Hazard ratios for all-cause death and first hospitalization within 2 years after HF for chronic heart failure patients with vs without moderate to severe depression.

|  | **All-Cause Death** | **Hospitalization** |
| --- | --- | --- |
| Number of patients | 425 | 425 |
| Number of events | 99 | 299 |
| Unadjusted HR (95% CI) | 2.45 (1.64-3.65) | 1.63 (1.25-2.14) |
| Adjusted* HR (95% CI) | 3.08 (1.92-4.92) | 1.66 (1.23-2.25) |

Moderate to severe depression defined as 9-item Patient Health Questionnaire (PHQ-9) ≥10.

*Adjusted for age, sex, systolic blood pressure, estimated glomerular filtration rate, blood urea nitrogen, serum sodium, elevated B-Type natriuretic peptide (BNP) or N-Terminal pro-BNP, ejection fraction, ischaemic aetiology and prior diabetes.

CI=Confidence interval; HR= Hazard Ratio.

**Table S3**

Comparison of the prognostic utility of adding moderate to severe depression to the base model in predicting all-cause mortality and hospitalization within 2 years after heart failure in chronic heart failure patients.

| Outcome | Model | AUC  (95% CI) | IDI, %  (95% CI) | NRI-continuous, % (95% CI) |
| --- | --- | --- | --- | --- |
| All-cause Death | Base model* | 0.781  (0.729-0.834) |  |  |
|  | Base model + depressive symptoms | 0.803  (0.751-0.855) | 4.88  (2.12-7.63) | 31.05  (12.46- 49.65) |
|  | p-value | 0.06 | 0.001 | 0.001 |
| Hospitalization | Base model* | 0.667  (0.609-0.724) |  |  |
|  | Base model + depressive symptoms | 0.687  (0.630-0.744) | 2.16  (0.96-3.36) | 21.34  (8.01-34.66) |
|  | p-value | 0.12 | <0.001 | 0.002 |

Moderate to severe depression defined as 9-item Patient Health Questionnaire (PHQ-9) ≥10.

*Base model includes age, sex, systolic blood pressure, estimated glomerular filtration rate, blood urea nitrogen, serum sodium, elevated B-Type natriuretic peptide (BNP) or N-Terminal pro-BNP, ejection fraction, ischaemic aetiology and prior diabetes.

AUC=area under curve; CI=confidence interval; IDI=integrated discrimination improvement; NRI=net re-classification improvement

**Part 2: Presence of depressive symptoms (PHQ-9 as a continuous variable) as a prognostic marker in HF patients**

**Table S4**

Hazard ratios for all-cause death and first hospitalization within 2 years after HF for chronic heart failure patients with 1 unit increase in PHQ-9.

|  | **All-Cause Death** | **Hospitalization** |
| --- | --- | --- |
| Number of patients | 425 | 425 |
| Number of events | 99 | 299 |
| Unadjusted HR (95% CI) | 1.08 (1.04, 1.11) | 1.05 (1.02, 1.07) |
| Adjusted* HR (95% CI) | 1.10 (1.06, 1.14) | 1.05 (1.02, 1.07) |

*Adjusted for age, sex, systolic blood pressure, estimated glomerular filtration rate, blood urea nitrogen, serum sodium, elevated B-Type natriuretic peptide (BNP) or N-Terminal pro-BNP, ejection fraction, ischaemic aetiology and prior diabetes.

CI=Confidence interval; HR= Hazard Ratio.
